# Hyperspectral Proximal Sensing of *Salix Alba* Trees in the Sacco River Valley (Latium, Italy)

**DOI:** 10.3390/s131114633

**Published:** 2013-10-29

**Authors:** Monica Moroni, Emanuela Lupo, Antonio Cenedese

**Affiliations:** DICEA-Sapienza University of Rome, via Eudossiana 18, 00184 Rome, Italy; E-Mails: emanuela.lupo@uniroma1.it (E.L.); antonio.cenedese@uniroma1.it (A.C.)

**Keywords:** hyperspectral imaging, environmental monitoring, proximal sensing

## Abstract

Recent developments in hardware and software have increased the possibilities and reduced the costs of hyperspectral proximal sensing. Through the analysis of high resolution spectroscopic measurements at the laboratory or field scales, this monitoring technique is suitable for quantitative estimates of biochemical and biophysical variables related to the physiological state of vegetation. Two systems for hyperspectral imaging have been designed and developed at DICEA-Sapienza University of Rome, one based on the use of spectrometers, the other on tunable interference filters. Both systems provide a high spectral and spatial resolution with low weight, power consumption and cost. This paper describes the set-up of the tunable filter platform and its application to the investigation of the environmental status of the region crossed by the Sacco river (Latium, Italy). This was achieved by analyzing the spectral response given by tree samples, with roots partly or wholly submerged in the river, located upstream and downstream of an industrial area affected by contamination. Data acquired is represented as reflectance indices as well as reflectance values. Broadband and narrowband indices based on pigment content and carotenoids *vs.* chlorophyll content suggest tree samples located upstream of the contaminated area are ‘healthier’ than those downstream.

## 1. Introduction

Hyperspectral remote and proximal sensing is a diagnostic tool that, in the context of sustainable land management and conservation, provides information about vegetation distributed in both space and time. Proximal sensing has interesting applications at small- and medium-scales. The analysis of high resolution spectroscopic measurements allows identification of features of the spectrum which provide a quantitative estimate of biochemical and biophysical variables related to the physiological state of the vegetation. The development of hyperspectral sensors at high spectral and spatial resolution then allows for seasonally or annually upgradeable field investigations, the identification of tree species, the mapping of vegetation cover, the understanding of biogeochemical cycles, and the detection of stress states [1,2].

Stress is defined as any environmental factor capable of inducing a potentially harmful physical or chemical change to plants. Stress can manifest itself in different forms and can be morphological or physiological. Stress detection helps identify which changes affect the vegetation in addition to the normal ones (for example related to seasonal cycles). Many stress events and a multitude of stressors exist in the life cycle of plants. Stressors may be grouped under natural or anthropogenic stress factors: high irradiance, extreme temperatures, pests and diseases, lack of water, herbicides, pesticides, fungicides, air pollution, nutrient deficiency or toxicity. Further, one has to differentiate between short-term and long-term stress effects as well as between low stress events, which can be partially compensated for by acclimation, adaptation and repair mechanisms, and strong stress or chronic stress events causing considerable damage that may eventually lead to cell and plant death [3]. The potential of proximal sensing in this field resides in the possibility of detecting a degenerative state of the vegetation in the area under examination. The spectral information may not be suitable for distinguishing among different states of stress because many causes may act simultaneously and different causes may produce similar effects on the plant physiology. Furthermore, vegetation monitoring must take into account factors which are not strictly stressors such as daily or seasonal cycles that alter the vegetation spectral response. Ground-truth verification is then required to confirm the presence and origin of a sensed stress state [4].

The applications of hyperspectral imaging to natural resources have been widely tested. We will review only few applications related to vegetation monitoring. The spectral signature of vegetation is influenced by the presence of pigments (mainly chlorophyll-a, chlorophyll-b, xanthophyll and carotenoids), whose content varies depending on the chemical and biological activity of the plant [5–7], the physical structure and water content of leaves [8]. The reflectance spectrum of a plant provides information on the degree of senescence, the deterioration of leaf structure or any diseases and abnormalities [5,8,9].

Sensing several wavelengths of the electromagnetic spectrum in the visible and near-mid infrared ranges, hyperspectral systems produce large data volumes which require the development of techniques and methods to handle these multi-dimensional datasets and reduce costs in data analysis and computer resources. The evaluation of appropriate hyperspectral indices both reduces the data dimensionality and helps understanding of the state of health of vegetation. In fact, the synthesis of appropriate indices allows one to drop redundant bands by the selection of optimal bands that capture most of the information on the target characteristics [10]. The computation of spectral vegetation indices implies a statistical approach employed to estimate vegetation biophysical characteristics from remotely sensed data. Otherwise a physical approach involving radiative transfer models describing the variation of canopy reflectance as a function of canopy, leaf and background characteristics can be employed [11]. Though both approaches have advantages and disadvantages, the statistical approach was used here since it is more consolidated and is then more appropriate for testing the hyperspectral imaging platform.

Many vegetation indices have been evaluated in an empirical way, generally through a combination of bands sensitive or not to stress. Indeed, the presence of stress is localized in specific wavelengths (feature positions) related to the physiological characteristics of plants. The verification of a change in certain bands can be a symptom of biological alteration. Vegetation indices are preferable than individual reflectance values to compensate for the effects of different lighting and weather conditions [12]. It is worth noting that accurate index calculation requires high quality reflectance measurements from hyperspectral sensors. Both broadband and narrowband indices have been employed to quantify pigment content and infer modification of the plant ecophysiological state and then correlate to the contamination of an area. Broadband indices have known limitations in providing adequate information on terrestrial ecosystem characteristics. This has led to an increasing interest in the narrowband indices which are expected to provide information that is more detailed [10]. Narrowband indices require careful attention to instrument calibration.

Table 1 presents the definition of the broadband and narrowband vegetation indices based on pigment content and carotenoids *vs.* chlorophyll content employed in this contribution. In the table, R refers to reflectance and the subscripts refer to specific spectral bands or wavelengths (*i.e.*, NIR refers to the average in the band interval 750–1,100 nm, VIS to the average in the band interval 580–750 nm, RED to the average in the band interval 600–700 nm and GREEN to the average in the band interval 500–600 nm; w refers to narrow wavelength at w nm).

Broadband vegetation indices are based on Green, Red, VIS and NIR bands, because vegetation exhibits unique reflectance properties in these bands which can be used to measure the development and stress state of a plant. Three types of indices have been developed, *i.e.*, simple ratios, differences, and normalised differences of reflectance values. Among broadband vegetation indices we have included:

- Ratio Vegetation Index (RVI)
- Difference Vegetation Index (DVI)
- Triangular Vegetation Index (TVI)
- Normalized Difference Vegetation Index (NDVI)
- Renormalized Difference Vegetation Index (RDVI)

RVI and NDVI have been widely used since the 1970s. NDVI and RVI, depending on infrared (region sensitive to the plant-leaf structure) and visible (region sensitive to the chlorophyll content) wavelengths, present values related to the photosynthetic capacity of the leaf. In general, high values indicate a better state: healthy vegetation absorbs most of the visible radiation and reflects a large portion of the infrared light. Instead, unhealthy plants reflect more in the visible and less in the infrared regions of the electromagnetic spectrum. Also DVI relates the reflectance at the infrared and visible wavelengths considering that the decrease at the former range is generally the most consistent response of vegetation to stress. TVI describes the radiative energy absorbed by the pigments as a function of the relative difference between RED and NIR reflectance in the green region, where the light absorption by chlorophyll is relatively insignificant [13]. It is expected to decrease for stressed vegetation. RDVI is a hybrid between DVI and NDVI and is supposed to combine the advantages of DVI and NDVI [13]. Healthy vegetation is expected to have higher values of RDVI.

Narrowband indices are based on selected wavebands associated with leaf pigments, whose variations may be related to the physiological state of leaves. Chlorophyll tends to decline more rapidly than carotenoids when plants are under stress or during senescence. Therefore narrowband indices relating bands sensitive to changes in chlorophyll content (550 nm and 700 nm) and insensitive bands (750 nm) have been introduced. In fact, it was found that for very low concentrations, the reflectance sensitivity is higher at the maximum absorption wavelength, located in the 675–700 nm region, and for medium-to-high chlorophyll concentrations reflectance sensitivity is higher around 550 nm [14]. One category of narrowband vegetation indices, based on pigment content, includes the indices below:

- NDVI_705_
- Narrow Band Ratios (NBRs)
- Pigment specific normalised difference (PSND)
- Pigment specific simple ratio (PSSR)

NDVI_705_ is a modified version of the NDVI. NDVI_705_ was introduced later as an improvement considering a narrow waveband at the edge of the chlorophyll absorption feature (e.g., 705 nm) rather than at the middle and referencing this against a waveband not influenced by chlorophyll content (750 nm), to capture the effect of varying chlorophyll contents [12].

Another category of narrowband vegetation indices has been derived considering that increases in the relative concentration of carotenoids with respect to chlorophyll are observed when plants are subjected to stress and in senescing leaves. Ratios of reflectance in the blue domain (where carotenoids and chlorophylls absorb) to the red domain (where only chlorophylls absorb) have been found to be highly correlated with this pigment ratio in different plant species, both at the leaf and canopy levels [14]. The narrowband vegetation index based on carotenoids *vs.* chlorophyll content is:

### Structure Insensitive Pigment Index (SIPI)

The SIPI index provides an estimate of the ratio of chlorophyll-a to carotenoids. The index, by introducing R_800_, a near-infrared band, minimizes the effects of radiation interactions at the leaf surface and internal structure of the mesophyll. Wavelengths 680 nm and 445 nm, empirically selected, correspond to the in-vivo absorption maxima of chlorophyll-a and carotenoids respectively [15]. The wavebands at 675, 650 and 500 nm, representing the absorption maxima of chlorophyll-a, chlorophyll-b and carotenoids respectively, have been replaced in these indices with 680 nm, 635 nm and 470 nm respectively, empirically determined by comparing spectral indices and pigment concentrations and measuring the correlation coefficient [5]. Spectral measurements from healthy vegetation are expected to provide higher values of the indices in this category.

Several investigators have related the changes in chlorophyll concentration to the shift in the Red Edge, *i.e.*, the inflection point that occurs in the rapid transition between red and infra-red reflectance at wavelengths around 720 nm [16]. The Red Edge position and shape, *i.e.*, blue-shift or red shift, are correlated with biophysical parameters at the canopy level and are considered additional indicators of stress [13]. The position of the Red Edge in the reflectance spectrum may be derived as the wavelength of the peak in the corresponding derivative spectrum between 680 nm and 750 nm, *i.e.*, the point of maximum slope.

At the Laboratory of Hydraulics of DICEA-Sapienza University of Rome, an effective methodology for hyperspectral monitoring has been developed. It is based on the use of two innovative experimental devices for acquiring hyperspectral images, one using interference filters, the other spectrometers. Both systems allow sampling the 400–1,800 nm spectral range with a spectral resolution higher than 10 nm. The system with spectrometers and an original algorithm for automatically combining multiple, overlapping images of a scene to form a single composition, have been employed in a proximal sensing field campaign conducted in San Teodoro (Olbia-Tempio—Sardinia). Mapping allowed for the identification of objects within the acquired image and agreed well with ground-truth measurements [17].

The systems have the following unique characteristics:

(1) Low cost compared to other systems available on the market;
(2) High spectral resolution;
(3) High spatial and temporal resolution;
(4) Easy portability, both systems have been engineered so that they can be transported by ultralight airplanes.

In this paper we present the data acquired during a proximal sensing field survey conducted in the valley of the Sacco river (Latium, Italy) with the interference filter platform. For proximal sensing field surveys with the equipment mounted on a fixed stand, the platform with tunable filters is preferred. This study is part of a larger project dedicated to the recovery of that area, subject in the last few years to a number of alarming cases of pollution (Environmental Quality & Territorial Protection–Sapienza JointLabs). The hyperspectral analysis was employed to detect the environmental status of the region crossed by the river. This was achieved by analyzing a number of spectral indices and waveband combinations derived from the spectral response of White Willow (*Salix Alba*) tree samples located upstream and downstream of an industrial area affected by contamination. A companion lab-scale study on vegetation subject to different types of stress (contamination with herbicides and pesticides, lack of water) was conducted with the same platform to validate the methodology at a smaller scale (results not shown).

The paper is organized as follows: Section 2 provides a review of most commonly employed vegetation indices; Section 3 illustrates the hyperspectral device with three interference filters; Section 4 describes the area under investigation; Section 5 presents the main steps for the analysis of hyperspectral data; Section 6 describes the results of the analysis. The paper ends with a concluding section.

## 2. Tunable Filter Based System

**Figure 1.**
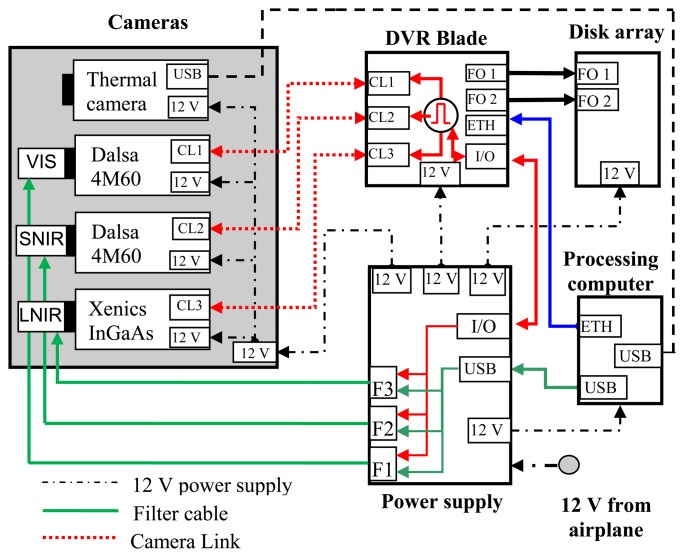
Apparatus with interference filters.

The system is based on the use of three Varispec interference tunable filters. Figure 1 shows the diagram of the system configuration, consisting of:

- One high-speed Digital Video Recorder (DVR) with three Camera Link inputs (IO Industries DVR Express® Blade) to acquire and manage the data from the cameras and to generate a trigger signal to tune the filter frequency and synchronize the camera acquisition;
- One 1-terabyte solid state disk array;
- One VIS filter mounted in front of a Dalsa 4M60 CMOS camera (2,352 × 1,728 pixels *@* 25 fps), hereinafter VIS system;
- One SNIR filter mounted in front of a Dalsa 4M60 CMOS camera (2,352 × 1,728 pixels *@* 25 fps), hereinafter SNIR system;
- One LNIR filter mounted in front of a Xeva Xenics InGaAS camera (640 × 512 pixels *@* 25 fps), hereinafter LNIR system;
- One thermal camera Cedip Jade UC;
- One power supply system for all devices;
- One processing computer for controlling the entire system and acquiring images of the thermal camera via a USB port (the thermal images are not discussed in this contribution).

**Figure 2.**
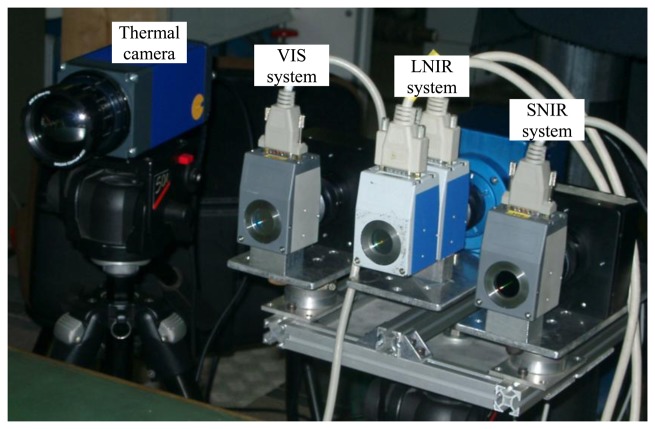
Picture of the hyperspectral apparatus: VIS system stands for Dalsa camera-VIS filter coupling; SNIR system for Dalsa camera-SNIR filter coupling and LNIR system for Xeva Xenics camera-LNIR filter coupling.

Figure 2 shows a picture of the hyperspectral apparatus equipped with the thermal camera. The first filter frequencies range between 400 nm and 720 nm (visible (VIS) filter), the second one between 650 nm and 1,100 nm (near infrared (SNIR) filter); the third one between 850 nm and 1,800 nm (mid infrared (LNIR) filter). The wavelength of transmitted light is electronically controllable through liquid crystal elements. The transmittance is not constant within the filter wavelength range. VIS filter transmittance increases with the wavelength; SNIR filter transmittance increases until 880 nm and then remains constant; LNIR filter transmittance oscillates tending to decrease for high wavelengths. Though the bandwidth for the VIS and SNIR filters is 10 nm and 6 nm for the LNIR filter, in order to simplify data acquisition and interpretation, all filters are tuned with a step of 10 nm.

**Figure 3.**
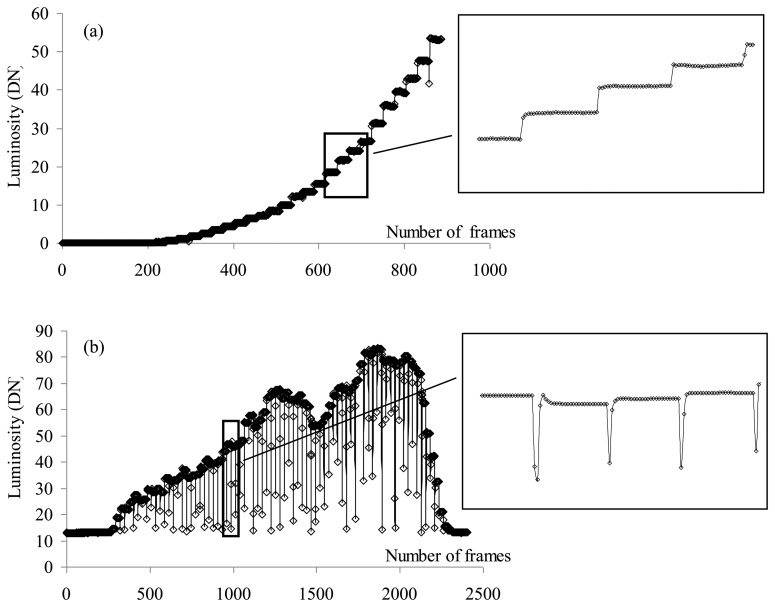
Luminosity trend within an acquisition cycle with filters (**a**) VIS and (**b**) LNIR.

When interference tunable filters were employed, it was necessary to acquire more pictures and tune each filter wavelength to gather the full spectrum of the scene. The cameras simultaneously acquired images at a rate of 25 frames per second and each filter was set to a given wavelength for one second. Roughly 25 images per wavelength were then available. The VIS system (filter + camera), acquiring images from 400 nm to 720 nm with 10 nm step, has acquired 25 images for each of the 33 bands, *i.e.*, approximately 825 images per cycle; the SNIR system, acquiring images for each of the 46 bands, has acquired about 1,150 images for each cycle; finally the LNIR system, tuned within 96 bands, has acquired about 2,400 images for each cycle. Since the filters have been tuned simultaneously and at least two cycles per filter were required, more VIS and SNIR cycles than LNIR cycles were acquired. Figure 3 presents the luminosity (in Digital Number DN) during an acquisition cycle for the VIS and LNIR filters. Due to the filter response time, *i.e.*, the low reliability of the device immediately after the selection of a new wavelength, the first images acquired at a given wavelength, especially for the LNIR filter, have to be rejected. Cameras were equipped with specifically designed achromatic lenses which minimise chromatic and spherical aberration effects.

The disk array was suitable for storing up to one hour of data acquisition by decreasing the resolution of the images acquired with the Dalsa cameras (set to 1,200 × 1,200 pixels). It is worth recalling that the system, which allows acquiring images without any compression, has been designed to reduce mass (less than 10 kg) and power consumption (less than 500 W power at start up).

## 3. Characterization of the Study Area

**Figure 4.**
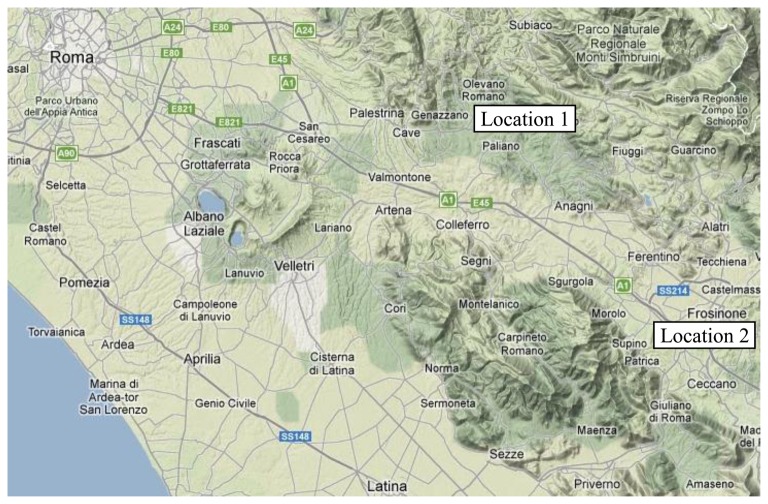
Map for localizing the area of study.

The Sacco river (latitude 41°31′00″ Nord, longitude 13°32′00″ Est), a sub basin of the Liri-Garigliano river, arises from Monte Casale, part of Monti Prenestini. Its waters cross the Province of Frosinone, near the town of Paliano, then flow into the Province of Rome through the municipalities of Genazzano, Valmontone and the city of Colleferro, and return to the province of Frosinone. The river continues toward the Latin valley, collecting waters of the tributaries from the Ernici and Lepini mountains and finally pouring its waters into the river Liri at Ceprano (Figure 4).

The Sacco river valley hosts numerous municipalities and is characterized by the presence of several industrial facilities (chemical, mechanical, electronic and food) and intense farming activities, mainly in the municipalities of Colleferro, Frosinone and Ceccano. One of the main issues for the environmental remediation of this territory concerns the assessed presence in soil and irrigation water of β-hexachlorocyclohexane (C_6_H_6_Cl_6_), a stable isomer of lindane, used as a pesticide until its prohibition.

Data were collected in three proximal sensing surveys, which took place in 2010, 2011 and 2012. The information concerning the measurement campaigns are reported in Table 2.

The field survey focused on two sections of the river: one (Location 1), in the municipality of Genazzano, is located upstream of the area that is supposed to be contaminated, the other (Location 2), in Ponte of Tomacella (Patrica-Frosinone), is located downstream (Figure 4). Both areas are characterized by a certain vegetation homogeneity with the predominance of White Willow (*Salix Alba*) and to a lesser extent of Black Poplar (*Populus Nigra*). The acquisition system was placed on the ground in both locations.

**Figure 5.**
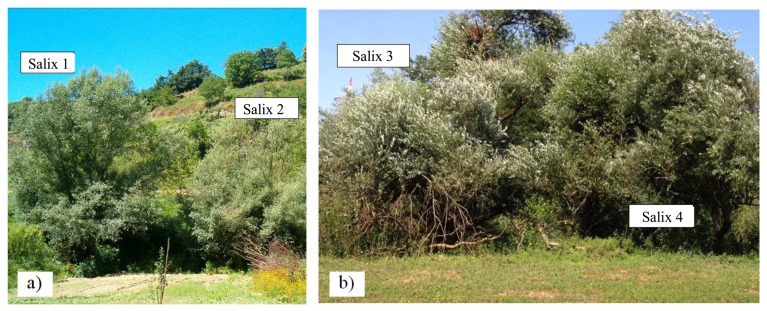
*Salix* monitored in Locations (**a**) 1 and (**b**) 2.

The entire tree canopy or a large part was included in the image. While no soil could be seen through the canopy, portions of sky are visible through the boundary of the canopy. The images were acquired as the sun was roughly at its zenith, in particular between 10 a.m. and 3 p.m., in order to provide maximum sunlight. The scene was orthogonal to the optics and at a distance of a few tens of meters. Figure 5a shows an image of the *Salix* trees monitored in Location 1, Figure 5b the *Salix* trees at Location 2. Hyperspectral images of both trees have been acquired within each field survey. Table 2 also reports the object employed for the radiometric calibration of the hyperspectral data.

## 4. Analysis of Hyperspectral Images

To construct the hyperspectral cubes, the acquired images were analyzed by specifically developed software and by commercial software (ENVI). The main steps of the analysis are listed below.

### Elimination of vignetting effects on each image

This imperfection, due to the camera lens and to the tunable filters placed in front of the camera, causes the reduction of brightness at image edges respect to its center. To account for the effects of the VIS and SNIR filters, the inner portion of the acquired images (1,200 × 1,200 pixels for the 4M60 cameras) was subject to processing. Eventual further vignetting effects were modelled by a cos^4^(α) fall-off in intensity away from the principal point, assuming that the optical axis passes through the image center [18]. The same law was applied to all spectral bands. Since only peripheral pixels presented a slight variation of the grey level (maximum 2 digital numbers), the consistency of the radiometric information is assured.

### Noise filtering

The CMOS and InGaAs arrays employed for image acquisition are subject to various sources of noise, including thermal, shot, and electronic noise in the amplified circuitry. While our cameras are equipped with 10 bit (Dalsa 4M60) and 14 bit (Xeva Xenics InGaAS) analog to digital converters, the system for image acquisition and storage allows managing 8-bit images. This may introduce a source of noise (usually termed intensity quantization) which, affecting low intensity value pixels, is not relevant within hyperspectral data analysis. To reduce noise effects, images have been convolved with a Gaussian mask. No appreciable effects, such as blur, have been noticed in the resulting images. The Xeva Xenics InGaAS camera is provided of software tools including bad pixel position and replacement. Bad pixels in the images have been removed prior to the Gaussian filter.

### Construction of the hyperspectral cube

The starting dataset for hyperspectral analysis is the hyperspectral cube, constructed by superimposing n images, namely cube bands, each representing the same spatial coordinates (the same area under investigation) but different radiometric information. Images at each wavelength have been extracted from the series acquired to build the hyperspectral cube in the VIS, SNIR and LNIR regions. Due to the synchronization of the filter tuning and the camera acquisition, sets of 25-frame-intervals may be associated with the given wavelengths, with the representative images being either one in the middle or the result of the average of a few images within each interval.

### Geometric transformation

Data have been acquired from three cameras. Then a geometric transformation (warping) of the hyperspectral cubes is required to place all the images on the same reference system and to build the hyperspectral cube of the entire wavelength range (400–1,800 nm). Some of the most common global transformations are related by a homography (similarity, affine, projective) or by polynomial functions [17]. Since the optical axes of the three cameras have been set almost parallel and the distance between the platform and the target was a few tens of meters, the area captured by the cameras was almost the same. A rotation, scaling, and translation warping (by selecting at least ground control points) was then adequate to improve the image to image correspondence. To avoid any influence on the pixel radiometric content, the nearest neighbor method was used to create the warped images.

### Radiometric calibration

This constitutes one of the most sensitive processing steps, since it ensures the construction of a spectral library as close as possible to the material characteristics. This is achieved by eliminating the dependence of the spectra on the measuring instruments (quantum efficiency of the sensor, filter transmission). In fact, the acquisition system does not record the material reflectance but rather the value of radiance, or that part of the reflected radiation that reaches the camera sensor with an energy content sufficient to be recorded. The absolute reflectance of the materials can be calculated only if the incident radiation on the target is known. In this case, the source of radiation being the Sun, it would be impossible to obtain the value of radiation incident on each point of the scene. The relative reflectance is then calculated. This is achieved by comparison with a reference spectrum chosen ad hoc. Two methods can be used: the Internal Average Relative Reflectance (IARR) and the Flat Field (FF). The first method derives correction parameters directly from the images while the second one, employed for this set of experiments, requires the presence of targets with smooth reference reflectance spectrum. A white reference standard (Spectralon) was then introduced in the scene for each acquisition. The Spectralon target was oriented consistently with the orientation of the tree canopy. The camera dark currents were also evaluated. Reflectance is calculated as follows: ((target-dark current)/(reference-dark current)). Spectral data obtained at ground level are mostly free of atmospheric effects [10]. So no further corrections were applied to the images.

## 5. Results

**Figure 6.**
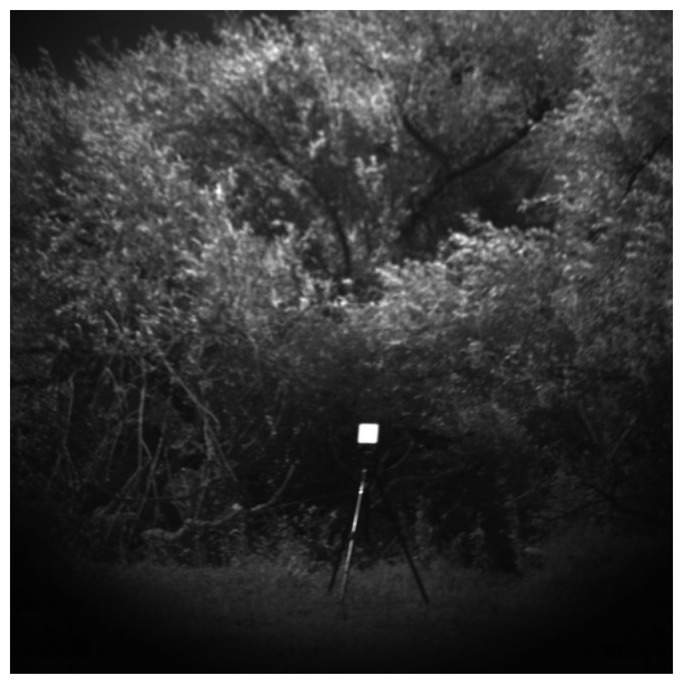
Image at wavelength 720 nm of the hyperspectral cube of *Salix* 4 in Location 2.

Figure 6 shows the image at the 720 nm wavelength of the hyperspectral cube of *Salix* 4 in Location 2 (each hyperspectral cube was built with 141 images ranging from wavelengths of 400 nm to 1,800 nm with a step of 10 nm). The reference standard (Spectralon) introduced in the scene for data radiometric calibration is also visible in Figure 6.

**Figure 7.**
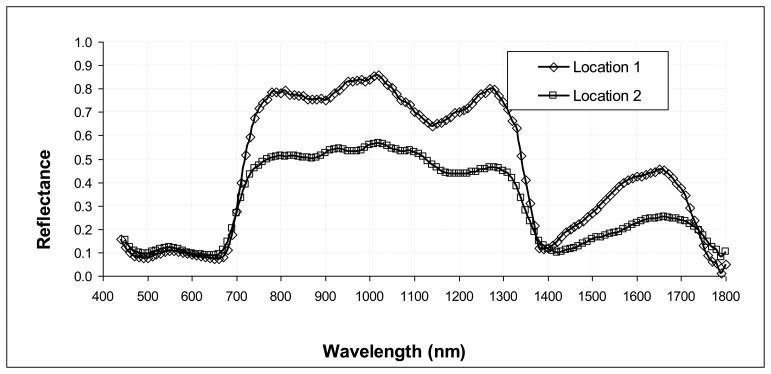
Representative reflectance spectra of *Salix* samples of Locations 1 and 2.

The average spatial resolution for the entire dataset was 0.28 cm/pixel. Tree samples at Locations 1 and 2 exhibit a characteristic behavior of the spectral reflectance in the range 400–1,800 nm, as illustrated in Figure 7. Since trees in both locations presented dense foliage, the spectral signatures have been derived by averaging the reflectance over an area comprising a large number of leaves without branches or background. The spectral signatures were substantial identical varying the dimension of the area, assuring only leaves were included. This ensures the radiometric calibration was properly performed.

Spectral reflectance of vegetation in the visible region of the electromagnetic spectrum is primarily governed by chlorophyll pigments. The pigments which absorb the greatest proportion of radiation in the visible region of the electromagnetic spectrum (400–700 nm) are chlorophyll-a and -b, which provide energy for photosynthesis, and carotenoids and anthocyanins which protect the reaction centers from excess of light and help intercept radiation in the visible range as auxiliary pigments of chlorophyll-a.

In the near infrared domain (700–1,300 nm) photosynthetic pigments do not present strong absorption features, so the magnitude of reflectance is governed by structural discontinuities encountered in the leaf and by the water content within the leaf, which, when stressed, undergoes an alteration in terms of internal distribution with a consequent significant variation in the spectral response. The spectral reflectance of vegetation in this region is characterized by very low reflectance in the red part of the spectrum followed by an abrupt increase in reflectance at 680–750 nm wavelengths. In the middle infrared region (1,300–1,700 nm), variable-reflectance values are mainly linked to the absorption characteristics of water and other compounds filtering part of the incident solar radiation which creates windows of absorption around 1,100 nm and 1,400 nm [14].

Both spectral signatures in Figure 7 reflect the typical behavior of vegetation described above, presenting two chlorophyll wells at the wavelengths of blue (480 nm) and red (660 nm), a green peak, which is not very clear although present, corresponding to the wavelength of green (roughly 550 nm), the red edge and NIR plateau. In the visible region, the comparison between the curves registers a slight increase of reflectance values for tree samples at Location 2. This is in accordance with [19], who observed that, for individual leaves, stress is associated with increased reflectance at visible wavelengths (400–700 nm).

In the near infrared region, from wavelength 700 nm, a significant reduction of reflectance, for *Salix* 3 of Location 2, occurs for all wavelengths detected. This confirms that digital imagery within key wavebands, particularly near 700 nm, could provide earlier detection of plant stress for most causes of stress and species [19].

Tables 3 and 4 present broadband and narrowband vegetation indices for the three field surveys and all *Salix* tree samples monitored. Due to technical problems, only *Salix* 1 in Location 1 and *Salix* 3 in Location 2 have been monitored during the second field survey.

Table 5 presents the average of the indices for each location computed using both *Salix* trees and data from all field surveys. Tree samples at Location 1 exhibit higher values of both broadband and narrowband vegetation indices. NDVI, NDVI_705_ and RDVI (most commonly used to detect vegetation stress states) of tree samples at Location 1 (average value of NDVI equal to 0.523, average value of NDVI_705_ equal to 0.371, average value of RDVI equal to 0.558) compared to tree samples at Location 2 (average value of NDVI equal to 0.412, average value of NDVI_705_ equal to 0.235, average value of RDVI equal to 0.393), indicate a better state of health of the vegetation in Location 1 compared to Location 2. NDVI_705_ presents a larger difference between Locations 1 and 2 compared to NDVI and RDVI. The difference in percentage of both TVI and DVI are remarkable. Also PSND_a_ and PSND_b_ and, to a larger extent, PSSR_a_ and PSSR_b_ present large differences. Presenting significant variations if computed from spectral data measured at Locations 1 rather than 2, all of these indices appear as good indicators of vegetation stress.

The red-edge, ranging between 700 and 720 nm for all tree samples regardless of their location, has been found to be a bad indicator of stress at the canopy level.

## 6. Conclusions

The set-up and improvement of a hyperspectral imaging system based on interference filters was proven during the described acquisition campaign. The system has several unique characteristics: a lower cost compared to other systems available on the market (roughly 130,000 €); high spectral resolution and high spatial and temporal resolution and portability. Sampling is rapid, portable, non-destructive, applicable to scales from leaf to canopy to stand and landscapes. The bandwidth is on the order of 10 nm, and the spatial resolution ranges up to the order of millimeters at the scale it was employed in the field survey, allowing great detail in extracted information.

The comparison of reflectance spectra and indices demonstrates the possibility of obtaining estimates of plant ecophysiological state. The measurements carried out show that the stress effects on the chemistry of the plant may occur beyond the visible region and, in particular, in the near infrared region. In other words, chlorosis, or yellowing of the leaf, can appear after other factors have occurred in a region of the spectrum not detectable by the human eye. The availability of a wide range of wavelengths is thus an indispensable element to highlight vegetation states of stress.

In field monitoring, variations in background reflectance properties, contributions from non-photosynthetic canopy components and the effects of leaf layering and canopy structure may weaken the relations between reflectance values in single wavebands and pigment concentrations. Vegetation indices which use ratios of reflectance at different wavelengths (especially those incorporating near-infrared wavebands) have been demonstrated to overcome such difficulties.

## Figures and Tables

**Table 1.** Broadband and narrowband vegetation indices. R refers to reflectance and the subscripts refer to specific spectral bands or wavelengths.

**Broadband Indices**	**Narrowband Indices**
$\text{RVI}=\frac{{\text{R}}_{\text{NIR}}}{{\text{R}}_{\text{VIS}}}$	$\begin{array}{c} \text{NBR1}=\frac{{\text{R}}_{750}}{{\text{R}}_{700}}\\ \text{NBR}2=\frac{{\text{R}}_{750}}{{\text{R}}_{550}} \end{array}$
$\text{DVI}={\text{R}}_{\text{NIR}}-{\text{R}}_{\text{VIS}}$	${\text{NDVI}}_{705}=\frac{{\text{R}}_{750}-{\text{R}}_{705}}{{\text{R}}_{750}+{\text{R}}_{705}}$
$\text{TVI}=0.5(120({\text{R}}_{\text{NIR}}-{\text{R}}_{\text{GREEN}})-200({\text{R}}_{\text{RED}}-{\text{R}}_{\text{GREEN}}))$	$\text{SIPI}=\frac{{\text{R}}_{800}-{\text{R}}_{445}}{{\text{R}}_{800}-{\text{R}}_{680}}$
$\text{NDVI}=\frac{{\text{R}}_{\text{NIR}}-{\text{R}}_{\text{VIS}}}{{\text{R}}_{\text{NIR}}+{\text{R}}_{\text{VIS}}}$	$\begin{array}{c} {\text{PSND}}_{\text{a}}=\frac{{\text{R}}_{800}-{\text{R}}_{680}}{{\text{R}}_{800}+{\text{R}}_{680}}\\ {\text{PSND}}_{\text{b}}=\frac{{\text{R}}_{800}-{\text{R}}_{635}}{{\text{R}}_{800}+{\text{R}}_{635}}\\ {\text{PSND}}_{\text{c}}=\frac{{\text{R}}_{800}-{\text{R}}_{470}}{{\text{R}}_{800}+{\text{R}}_{470}} \end{array}$
$\text{RDVI}=\sqrt{\text{NDVI}\times \text{DVI}}$	$\begin{array}{c} {\text{PSSR}}_{\text{a}}=\frac{{\text{R}}_{800}}{{\text{R}}_{680}}\\ {\text{PSSR}}_{\text{b}}=\frac{{\text{R}}_{800}}{{\text{R}}_{635}}\\ {\text{PSSR}}_{\text{c}}=\frac{{\text{R}}_{800}}{{\text{R}}_{470}} \end{array}$

**Table 1.** Dates of the Sacco river field survey.

**Field Survey**	**Date**	**Location**		**Calibration Object**
1	1 October 2010	1	2	Polystyrene
2	11 October 2011	1	2	Spectralon
3	25 June 2012	1	2	Spectralon

**Table 3.** Broadband vegetation indices.

**Field Survey**	**Location**	***Salix* Tree**	**Broadband Indices**				

			**RVI**	**DVI**	**TVI**	**NDVI**	**RDVI**
1	1	1	3.232	0.613	46.390	0.527	0.569
1	1	2	2.927	0.532	39.559	0.491	0.511
1	2	3	2.581	0.377	25.768	0.442	0.408
1	2	4	1.711	0.261	17.509	0.262	0.262
2	1	1	3.245	0.604	45.904	0.529	0.565
2	2	3	2.410	0.423	29.960	0.414	0.418
3	1	1	3.282	0.546	39.876	0.533	0.539
3	1	2	3.307	0.690	49.309	0.536	0.608
3	2	3	2.602	0.323	23.500	0.445	0.379
3	2	4	2.573	0.418	31.173	0.440	0.429

**Table 2.** Narrowband vegetation indices.

**Field** **Survey**	**Location**	***Salix*** **Tree**	**Narrowband Indices**									
			**NDVI_705_**	**NBR1**	**NBR2**	**PSND**_a_	**PSNDb**	**PSND**_c_	**PSSR**_a_	**PSSR**_b_	**PSSR**_c_	**SIPI**
1	1	1	0.421	3.077	4.620	0.739	0.799	0.615	6.670	8.950	4.199	0.750
1	1	2	0.300	2.147	5.436	0.649	0.797	0.640	4.704	8.832	4.556	0.838
1	2	3	0.179	1.600	2.550	0.520	0.743	0.814	3.163	6.797	9.755	1.255
1	2	4	0.097	1.313	2.772	0.305	0.460	0.610	1.879	2.702	4.123	1.391
2	1	1	0.352	2.528	3.348	0.808	0.758	0.761	9.409	7.257	7.361	0.849
2	2	3	0.268	1.889	2.527	0.500	0.548	0.531	3.040	3.442	3.391	0.762
3	1	1	0.360	2.581	6.649	0.750	0.815	0.802	6.988	9.827	9.123	0.957
3	1	2	0.425	2.939	4.458	0.706	0.735	0.695	5.796	6.547	5.556	0.893
3	2	3	0.227	1.785	4.262	0.739	0.726	0.671	3.586	6.338	5.086	0.973
3	2	4	0.291	2.111	3.737	0.617	0.668	0.639	4.252	5.098	4.571	0.895

**Table 5.** Vegetation index average values and differences between Locations 1 and 2.

**Vegetation Index**		**Average Value at** **Location 1**	**Average Value at** **Location 2**	**Index Difference between** **Locations 1 and 2 (%)**
**Broadband Indices**	RVI	3.199	2.432	31.5
	DVI	0.597	0.377	58.3
	TVI	44.208	27.179	62.7
	NDVI	0.523	0.412	27.0
	RDVI	0.558	0.393	41.9
**Narrowband Indices**	NBR_1_	2.654	1.846	43.8
	NBR_2_	4.902	3.903	25.6
	NDVI_705_	0.371	0.235	58.2
	PSND_a_	0.730	0.534	36.7
	PSND_b_	0.781	0.640	22.0
	PSND_c_	0.703	0.649	8.3
	PSSR_a_	6.714	3.489	92.4
	PSSR_b_	8.283	4.939	67.7
	PSSR_c_	6.159	5.153	19.5
	SIPI	0.858	1.009	−15.0

## References

[1] Zarco-Tejada P.J., Miller J.R., Mohammed G.H., Noland T.L., Sampson P.H. (2002). Vegetation stress detection through chlorophyll a + b estimation and fluorescence effects on hyperspectral imagery. J. Environ. Qual..

[2] Xie Y., Sha Z., Yu M. (2008). Remote sensing imagery in vegetation mapping: A review. J. Plant Ecol..

[3] Lichtenthaler H.K. (1996). Vegetation stress: An introduction to the stress concept in plants. J. Plant Physiol..

[4] Haboudane D., Miller J.R., Tremblay N., Zarco-Tejada P.J., Dextraze L. (2002). Integrated narrow-band vegetation indices for prediction of crop chlorophyll content for application to precision agriculture. Remote Sens. Environ..

[5] Blackburn G.A. (1998). Spectral indices for estimating photosynthetic pigment concentrations: A test using senescent tree leaves. Int. J. Remote Sens..

[6] Sims A.D., Gamon J.A. (2003). Estimation of vegetation water content and photosynthetic tissue area from spectral reflectance: A comparison of indices based on liquid water and chlorophyll absorption features. Remote Sens. Environ..

[7] Sims A.D., Gamon J.A. (2002). Relationships between leaf pigment content and spectral reflectance across a wide range of species, leaf structures and developmental stages. Remote Sens. Environ..

[8] Baret F., Fourty T. (1998). Estimation of leaf water content and specific leaf weight from reflectance and transmittance measurements. Agronomie.

[9] Carter G.A., Miller R.L. (1994). Early detection of plant stress by digital imaging within narrow stress-sensitive wavebands. Remote Sens. Environ..

[10] Thenkabail P.S., Smith R.B., De-Pauw E. (2002). Evaluation of narrowband and broadband vegetation indices for determining optimal hyperspectral wavebands for agricultural crop characterization. Photogramm. Eng. Remote Sens..

[11] Darvishzadeh R., Atzberger C., Skidmore A., Schlerf M. (2011). Mapping grassland leaf area index with airborne hyperspectral imagery: A comparison study of statistical approaches and inversion of radiative transfer models. ISPRS J. Photogramm. Remote Sens..

[12] Gamon J.A., Surfus J.S. (1999). Assessing leaf pigment content and activity with a reflectometer. New Phytol..

[13] Broge N.H., Leblanc E. (2000). Comparing prediction power and stability of broadband and hyperspectral vegetation indices for estimation of green leaf area index and canopy chlorophyll density. Remote Sens. Environ..

[14] Peñuelas J., Filella I. (1998). Visible and near-infrared reflectance techniques for diagnosing plant physiological status. Trends Plant Sci..

[15] Peñuelas J., Baret F., Filella I. (1995). Semi-empirical indices to assess carotenoids/chlorophyll a ratio from leaf spectral reflectance. Photosynthetica.

[16] Gitelson A., Merzlyak M., Lichtenthaler H. (1996). Detection of red edge position and chlorophyll content by reflectance measurements near 700 nm. J. Plant Physiol..

[17] Moroni M., Dacquino C., Cenedese A. (2012). Mosaicing of hyperspectral images: The application of a spectrograph imaging device. Sensors.

[18] Klein M.V., Furtak T.E. (1986). Optics.

[19] Carter G.A. (1994). Ratios of leaf reflectances in narrow wavebands as indicators of plant stress. Int. J. Remote Sens..

